# Enhancing environmental policy through evidence synthesis: a review of the Environmental Evidence for the Future (EEF) Initiative

**DOI:** 10.1186/s13750-024-00329-2

**Published:** 2024-03-29

**Authors:** Kathryn Anne Monk

**Affiliations:** Department of Biosciences, Swansea University, Singleton Park, Swansea, SA2 8PP UK

**Keywords:** Evidence synthesis, UK environmental policy, Systematic mapping, Stakeholder engagement, Collaboration, Brexit impacts, Thematic prioritization, Sustainability challenges, Evidence-based decision making, Interdisciplinary research

## Abstract

The Environmental Evidence for the Future (EEF) Initiative emerged in response to the challenges and opportunities presented by the UK’s decision to leave the European Union and its associated Environmental Frameworks. The Natural Environment Research Council (NERC), working closely with the Collaboration for Environmental Evidence (CEE) and UK stakeholders, developed the initiative to identify and address crucial evidence gaps, offering a long-term vision for environmental policy and sustainability. The EEF Initiative progressed through three stages: strategic priority identification, NERC panel award selection, and the production of Systematic Maps of existing evidence. The first stage involved collaborative workshops across the UK to identify key knowledge gaps in environmental science. The subsequent prioritisation resulted in 65 challenges across 10 thematic areas. The second stage saw NERC initiating, with CEE support, an open call for research proposals emphasising the use of evidence synthesis methodology. The selection process, balancing topic importance and applicant expertise, led to funding for five projects. The final stage involved the production of Systematic Maps of existing evidence based on the CEE Guidelines and Standards, providing a structured overview of existing literature on specific topics. The EEF Initiative demonstrated effective collaboration between UKRI (NERC), an independent non-profit (CEE), academia, and government agencies, addressing critical environmental challenges through rigorous evidence synthesis methodologies. The programme enhanced understanding and utilisation of these methodologies within the research community. Key lessons include the importance of inclusive priority-setting, differentiation between broad policy questions and specific Systematic Map questions, recognition of the value of Systematic Maps, and the role of experience in evidence synthesis teams. As policymakers and researchers navigate environmental policies in a resource-constrained environment, the EEF Initiative highlights the cost-effectiveness and efficiency of systematic mapping and review processes for evidence-based decision-making. The success of funding through NERC sets a precedent for future thematic evidence focused programmes, emphasising the need for continued support in developing synthesis skills among researchers and encouraging direct government commissions for targeted and responsive evidence. The EEF Initiative serves as a model for effective collaboration, providing valuable insights into addressing evidence gaps and shaping evidence-based policymaking in an ever-evolving environmental landscape.

## Introduction

The Environmental Evidence for the Future (EEF) Initiative represented an innovative and significant collaboration between the Natural Environment Research Council (NERC)^1^ and the international non-profit Collaboration for Environmental Evidence (CEE), as well as with key UK government departments, regulatory agencies, and other non-governmental organisations. The core objective of the initiative was to address crucial evidence gaps emerging in the wake of the UK’s decision to leave the European Union (EU) and its associated Environmental Frameworks. This departure brought substantial challenges and opportunities for environmental policy and sustainability.

As explained in the NERC Announcement of Opportunity for the EEF Initiative, the EU Frameworks had served as crucial benchmarks for UK scientists, guiding their studies to inform policymaking and implementation in such areas as agriculture and waste management. The UK consequently faced decisions around environmental policy, legislation and regulation, some of which were required to be made on very rapid timescales. The UK also, however, had a unique longer-term opportunity to take a fresh and innovative approach to developing and implementing policy and to the provision of evidence to inform that process. In addition, each of the UK devolved administrations (the Scottish Government, the Welsh Government, and the Northern Ireland Executive), has varying degrees of legislative power, with Scotland and Wales having their own parliaments and governments, while Northern Ireland has a power-sharing executive. In terms of environmental policy decision-making and execution, the devolved nations can create their own environmental policies and legislation in areas that have been devolved to them, such as agriculture, forestry, and the environment. Certain areas, such as energy and some aspects of environmental regulation, however, remain reserved to the UK Parliament. The EEF Initiative emerged from the need to build on and strengthen the common environmental evidence base in the long-term, by:

- Working in close collaboration with relevant UK and devolved administration policy makers and agencies to identify and define crucial environmental policy challenges and opportunities that present from the UK leaving the EU;
- Enabling academic freedom to propose innovative ways to inform decisions and pioneer innovative policies and solutions in response to these challenges and opportunities.

Prioritising these efforts on critical areas, enabling decision makers to use evidence systematically and transparently with possibly decreasing funds, was essential. As such, a unique characteristic of the EEF Initiative compared to the usual NERC funded programmes, was the requirement for proposals to use Systematic Mapping as a research method. Systematic Maps provide a comprehensive and structured overview of existing literature and evidence on a specific topic, summarising key characteristics of studies, such as their geographic distribution, methodologies, and subject focus, without conducting a full Systematic Review that would synthesise findings. The Systematic Maps produced under the EEF Initiative would therefore identify the current relevant research landscape and gaps in knowledge and inform future Systematic Reviews or primary research efforts.

In this paper, I examine three distinct stages of the EEF Initiative: the identification of strategic priorities through a Programme Advisory Group and workshops, the NERC panel process for award selection, and the production of the evidence syntheses through Systematic Maps. I also explore lessons learnt from this process.

### Stage 1: Identifying strategic priorities

The first stage of the EEF Initiative, aimed to define and prioritise key knowledge gaps in the environmental science evidence base, was delivered by the UK Centre for Ecology and Hydrology (UKCEH) under contract to NERC. This process involved close collaboration with stakeholders from various sectors. Whilst government was key, participants were also sought from civil society organisations and businesses. A crucial method employed in this process was the organisation of four UK-wide futures-focussed workshops held between August and September 2017 in Scotland, Wales, Northern Ireland, and England (encompassing perspectives from the UK Overseas Territories). The aim of the workshops was to explore the long-term opportunities and challenges for environmental policy in light of leaving the EU. The approach recognised the different policy and governance contexts across the devolved administrations and the need to seek a broad range of perspectives. Following the workshops, UKCEH produced a set of 100-word ‘future environmental policy and practice challenge’ areas which were shared via an open call to the community to articulate the key knowledge gaps and evidence needs pertaining to the challenges identified. This took place over a four-week period in late 2017. The working undertaken during this ‘discovery’ phase revealed insights and challenges associated with environmental policy in a ‘post-Brexit’ landscape.

The outcome of this initial stage was a comprehensive report compiled by UKCEH [8]. The report methodically outlined the priorities that would guide the subsequent phases of the EEF Initiative. It identified a final set of 65 “100-word challenges” clustered within 10 thematic areas: Land and Marine Use, Climate Change, Economics of Resource Use, Soils, Biodiversity, Environmental Policy, Human Health, Technology, Circular Economy, and International Focus. Emphasising interdisciplinary and transdisciplinary research, the report underscored the critical need to integrate diverse datasets to inform future environmental policymaking and practice.

Key issues at this stage included ensuring sufficient representation from the different stakeholder groups and a sufficient level of awareness across all participants of futures thinking and prioritisation processes to enable effective debate.

### Stage 2: The NERC panel process for award selection

Using the structure of the UKCEH analyses of priorities, NERC initiated an open call for the EEF Initiative via the standard NERC grant award system, with modifications provided by CEE to ensure the use of evidence synthesis methodology. Within this framework, applicants from the research community submitted proposals designed to tackle the prioritised thematic challenges. The core objective was to employ systematic mapping and review processes to address these challenges. This focus on evidence synthesis, the requirement for a specific methodology and standards to be used, and the involvement of a non-profit organization, CEE, to guarantee its effective implementation, was unique for NERC. The UK Research Councils that routinely fund rigorous evidence synthesis programmes are mainly the National Institute for Health Research (NIHR) and the Economic and Social Research Council (ESRC).

The NERC assessment panel for the call was characterised by a diversity of backgrounds and perspectives. It consisted of policymakers, representatives from environmental regulatory bodies, and academic experts in evidence synthesis, and was chaired by CEE. This diversity of membership brought different lenses to the evaluation process with, in general, policymakers focusing on the subject matter and academics emphasising the quality and experience of the applicants.

One of the principal challenges during this stage lay in achieving a delicate balance between the importance and potential impact of the research topic and the expertise of the applicants in effectively conducting systematic maps. Additionally, there was considerable variation in levels of awareness and understanding in both the research and user communities concerning the significance of Systematic Maps when compared to less rigorous and less repeatable review approaches that are more traditional. It was also crucial that assessors clearly differentiated between a broad policy question and a question appropriate for a Systematic Map, as this distinction significantly affected the success of this stage.

Ultimately, the selection process led to the funding of five projects, each tasked with producing a Systematic Map protocol and final report designed to address a specific issue within one of the thematic areas identified in the prioritisation stage (Table 1).

**Table 1:** Grants awarded in the delivery phase of the EEF initiative

Grant principal investigator	Organisations	Project title	Protocols and final publications
Dr Alexandra Collins	Imperial College London	What are the impacts of agricultural soil and crop management on greenhouse gas fluxes? – Informing post Brexit agricultural subsidy policy	[2, 3]
Dr Katherine Yates	University of Salford York University	Evidence synthesis to inform monitoring and evaluation of marine spatial management in the UK	[9, 10]
Dr Ruth Garside	University of Exeter	Mapping the evidence for the risks of human exposure and transmission of AMR in the natural environment	[11, 12]
Dr Jeremy Graham Carter	The University of Manchester	Identifying and prioritising nature based climate change adaptation measures for addressing future flood risk: creating a systematic evidence map	[4, 5]
Dr Jan Dick	CEH The James Hutton Institute University of Leeds National Trust/University of Exeter	Evidence for nature based solutions (NBSGap)	[6, 7]

### Stage 3: Production of systematic maps

The production of the EEF Systematic Maps entailed various approaches and timelines, as each project team encountered different challenges. Two progress meetings, jointly organised by NERC and CEE, were held, the first to provide feedback on the protocols and the second to provide training and advice for less experienced teams. Some teams exhibited swifter progress due to their experience and the precision of their research questions. In contrast, others encountered difficulties stemming from initially less focused questions and the intricacies of complex searches. These challenges underscored the value of leveraging the Collaborative for Environmental Evidence (CEE) Guidance and Standards (latest version: CEE [1]), particularly for less experienced teams.

All five Systematic Maps were subsequently completed through the CEE process in accordance with their Guidance and Standards. All protocols and Systematic Maps were published as a collection in the journal *Environmental Evidence* (https://www.biomedcentral.com/collections/en-ev-policy). Notably, the EEF Initiative funding had included the cost of publication fees. Table 2 gives an indication of interest in the Systematic Maps, citing accesses and citations to date as reported by the journal. Comparing the findings of the Systematic Maps provided valuable insights into the state of existing knowledge within various environmental domains (Table 2). These maps serve as essential tools for policymakers and other environmental managers and concerned parties, offering a structured and comprehensive overview of the evidence landscape. Consequently, they should play a crucial role in guiding strategic decisions.

**Table 2:** Summaries of findings of the five systematic maps produced through the EEF Initiative

Grant principal investigator	Summary of findings of systematic map	Systematic Map online accesses/citations as of mid February 2024/year of publication^a^
Dr Alexandra Collins	Investigates the impact of farmland management practices on greenhouse gas emissions in temperate regions, stressing the need for comprehensive research considering diverse practices and regional variations	2629 / 2 / 2022
Dr Katherine Yates	Focuses on methodologies for monitoring marine spatial management measures, revealing gaps in assessing social and economic impacts and the need for comprehensive frameworks in different environmental contexts	5366 / 8 / 2021
Dr Ruth Garside	Addresses antibiotic resistance transmission from environmental sources to humans, emphasizing the need for a deeper understanding of health impacts and exploring various transmission pathways beyond consumption/ingestion	10 k / 31 /2022
Dr Jeremy Graham Carter	Examines the effectiveness of Natural Flood Management (NFM) in the UK, revealing gaps in understanding and emphasizing the importance of integrating climate change adaptation in NFM strategies	1915 / 1 /2023
Dr Jan Dick	Explores the link between nature-based solutions (NBS) and human well-being in the UK, highlighting biases in research and emphasizing the necessity for robust, long-term studies comparing NBS with non-NBS alternatives across various societal challenges	6649 / 16 / 2020

^a^It is important to note that number of citations depends heavily on time since publication. This varies between 2020 and 2023 (see Table 1)

### Follow-up actions

Authors from all five projects shared feedback on their key follow-up actions undertaken with end-users after the completion of their projects. Focus on these has varied, some having closer connections with specific stakeholders than others. A significant factor was whether the projects had the time and funds available after completion of the maps themselves to undertake continued engagement with end users. There had also been an intention to convene an overall end-of-programme stakeholder event, but challenges and delays related to the Covid-19 pandemic in particular meant that this did not happen. Examples of follow-up activities undertaken by the Systematic Map authors included:

1. Continuing stakeholder engagement through workshops, ongoing collaboration, and the incorporation of project outcomes into various research outputs, highlighting the practical significance of the research.
2. Providing recommendations for allocating resources to address evidence gaps and stakeholder needs, underlining the potential for building upon the initial research with additional funding.
3. Serving as a foundation for further academic work, with one author highlighting a Ph.D. student using their project’s outcomes to conduct a Systematic Review, illustrating how research can have a lasting impact by contributing to ongoing scholarly investigations in the field and the enhancing of the broader evidence base for decision making.

### Lessons learned

Summarising informal discussions with Initiative participants and NERC, and a review of the NERC Advisory Group minutes, the EEF Initiative offers five important lessons that may guide both researchers and policymakers involved in similar future evidence synthesis activities designed to inform environmental policymaking:

1. Inclusivity in Priority Setting: The importance of inclusive engagement during the priority-setting phase cannot be overstated. To ensure a well-rounded understanding of knowledge gaps and evidence needs, it is essential to involve stakeholders from a broad range of sectors and backgrounds. Stakeholders themselves need to recognise the significance of their individual focus as well as their understanding of the processes involved.
2. Differentiating Policy and Systematic Map Questions: Clear differentiation between broad policy questions and specific questions suitable for systematic mapping or other rigorous evidence synthesis methods is crucial. Policymakers and researchers must collaborate effectively to formulate precise research questions that reflect evidence needs that align with policy goals and recognise that sufficient time and team resilience must be factored into the synthesis timeline. The new UK Economic and Social Research Council and UK Government Office of Science funded Areas of Research Interest (ARI) database should be a valuable tool here. It pulls together in one place all UK government department ARIs (with plans to expand its content to include research and evidence needs across the devolved administrations): https://ari.org.uk.
3. Recognizing the Value of Systematic Maps: Systematic Maps play a unique role in objectively and repeatably summarizing existing evidence, identifying research priorities, and guiding future evidence synthesis. It is essential to enhance recognition of their value, and that of more focussed syntheses such as Systematic Reviews and Rapid Assessments, within both the academic and policymaking communities, and essential for any members of relevant grant selection panels. Again, for the UK, the ARIs database may help here to avoid unnecessary primary research, as a large proportion of the ARIs could be addressed through evidence synthesis.
4. The Role of Experience: Experience in evidence synthesis teams significantly influences the efficiency and quality of systematic mapping projects. Recognising and supporting less experienced teams is critical for their success and the general improvement of evidence-informed decision making. Increasing awareness and capacity within the research community more broadly to use these methods is also necessary, through specific training opportunities and developing better networks between more experienced teams and early career researchers.
5. The Role of Research Funding Bodies versus Government Direct Funds for Evidence Synthesis for User Decision Making: Research funding bodies such as UKRI and its Research Councils provide a route through which researchers can access the learning/ training on how to undertake evidence synthesises as well as directly funding evidence syntheses that provide impartial research but may lack customisation and responsiveness to immediate policy needs. Consideration should be given to the availability of rapid response funds and support for closer collaboration with policy partners. Government-contracted syntheses offer tailored research directly aligned with government priorities, promoting responsiveness, transparency, and efficiency. The choice between the two depends on the specific needs of policymaking and the balance between impartiality and policy relevance.

## Conclusions

The Environmental Evidence for the Future (EEF) Initiative was an innovative example of effective funding and collaboration between NERC and an independent non-profit, along with academia and government agencies, in addressing critical environmental challenges by using rigorous evidence syntheses methodologies. Through the collaborative identification of priorities and grant selection processes, and the subsequent production of Systematic Maps, the programme has demonstrated how rigorous, open and transparent contributions to evidence-based policymaking in the UK, and possibly in other countries, can be shaped and delivered.

The EEF Initiative has played an important role in increasing the understanding and use of these methodologies in the research community, and highlighted some of the considerations to be taken into account by the user community when deciding on the funding mechanism most appropriate to the delivery of the evidence they need.

As policymakers and researchers increasingly work together to shape environmental policies, the lessons learned from the EEF Initiative highlight the importance of evidence synthesis in guiding strategic decisions and, in an environment of decreasing funds and staff time for commissioning new evidence, ensuring that resources are well deployed. Systematic mapping and review processes can provide a cost-effective, efficient, and evidence-based approach to inform decision-making while identifying gaps for targeted primary research when necessary. Funding these through NERC in this demonstration activity has set a precedent that can be built on through further thematic funding programmes, through the focussed development of synthesis skills in post-graduate and early career researchers, and through support for directly commissioned government work.

## Data Availability

Not applicable.

## Footnotes

1 NERC is part of UK Research and Innovation (UKRI).

## References

[1] Collaboration for Environmental Evidence. 2022. Guidelines and Standards for Evidence synthesis in Environmental Management. Version 5.1 (AS Pullin, GK Frampton, B Livoreil & G Petrokofsky, Eds) www.environmentalevidence.org/information-for-authors.

[2] Collins AM, Haddaway NR, Macura B, et al. What are the impacts of within-field farmland management practices on the flux of greenhouse gases from arable cropland in temperate regions? A systematic map protocol. Environ Evid. 2019;8:38. 10.1186/s13750-019-0182-2. 10.1186/s13750-019-0182-2 PMC11378830 39294834

[3] Collins AM, Haddaway NR, Thomas J, et al. Existing evidence on the impacts of within-field farmland management practices on the flux of greenhouse gases from arable cropland in temperate regions: a systematic map. Environ Evid. 2022;11:24. 10.1186/s13750-022-00275-x. 10.1186/s13750-022-00275-x PMC11378830 39294834

[4] Connelly A, Snow A, Carter J, et al. What approaches exist to evaluate the effectiveness of UK-relevant natural flood management measures? A systematic map protocol. Environ Evid. 2020;9:11. 10.1186/s13750-020-00192-x. 10.1186/s13750-020-00192-x PMC11378772 39294822

[5] Connelly A, Snow A, Carter J, et al. What approaches exist to evaluate the effectiveness of UK-relevant natural flood management measures? A systematic map. Environ Evid. 2023;12:12. 10.1186/s13750-023-00297-z. 10.1186/s13750-023-00297-z PMC11378772 39294822

[6] Dick J, Miller JD, Carruthers-Jones J, et al. How are nature based solutions contributing to priority societal challenges surrounding human well-being in the United Kingdom: a systematic map protocol. Environ Evid. 2019;8:37. 10.1186/s13750-019-0180-4. 10.1186/s13750-019-0180-4

[7] Dick J, Carruthers-Jones J, Carver S, et al. How are nature-based solutions contributing to priority societal challenges surrounding human well-being in the United Kingdom: a systematic map. Environ Evid. 2020;9:25. 10.1186/s13750-020-00208-6. 10.1186/s13750-020-00208-6

[8] Dick J, Gray A, Chamberlain J, Bealey W, Garbutt A, Evans A, Lacy H, Burns P, Hails R. 2018. Environmental Evidence for the Future Initiative Final Report. CEH Ref: NEC06314. Confidential report held by CEH.

[9] O’Leary BC, Stewart BD, McKinley E, et al. What is the nature and extent of evidence on methodologies for monitoring and evaluating marine spatial management measures in UK and similar coastal waters? A systematic map protocol. Environ Evid. 2019;8:34. 10.1186/s13750-019-0178-y. 10.1186/s13750-019-0178-y

[10] O’Leary BC, Copping JP, Mukherjee N, et al. The nature and extent of evidence on methodologies for monitoring and evaluating marine spatial management measures in the UK and similar coastal waters: a systematic map. Environ Evid. 2021;10:13. 10.1186/s13750-021-00227-x. 10.1186/s13750-021-00227-x

[11] Stanton IC, Bethel A, Leonard AFC, et al. Existing evidence on antibiotic resistance exposure and transmission to humans from the environment: a systematic map. Environ Evid. 2022;11:8. 10.1186/s13750-022-00262-2. 35308196 10.1186/s13750-022-00262-2 PMC8917330

[12] Stanton IC, Bethel A, Leonard AFC, et al. What is the research evidence for antibiotic resistance exposure and transmission to humans from the environment? A systematic map protocol. Environ Evid. 2020;9:12. 10.1186/s13750-020-00197-6. 32518638 10.1186/s13750-020-00197-6 PMC7268584

